# Combined hormonomic, transcriptomic, and metabolomic analyses reveal the role of programmed cell death in bud abscission of Chinese chestnut (*Castanea mollissima* BL.)

**DOI:** 10.3389/fpls.2026.1781873

**Published:** 2026-04-07

**Authors:** Yan Guo, Shuhang Zhang, Ying Li, Xinfang Zhang, Jinyu Liu, Jiayi Liu, Liying Fan, Mengfan Qin, Guangpeng Wang

**Affiliations:** 1 Changli Institute of Pomology, Hebei Academy of Agricultural and Forestry Sciences, Qinhuangdao, China; 2 Crop Research Institute, Guangdong Academy of Agricultural Science, Guangzhou, China

**Keywords:** autophagy, bud, chestnut, multi-omics, programmed cell death

## Abstract

**Introduction:**

The programmed cell death of buds directly affects the formation of a dwarf and compact crown in chestnut (*Castanea mollissima* BL.), making it an agronomically important trait; however, the molecular and metabolic regulatory mechanisms underlying this process remain unclear.

**Methods:**

This study used ‘the abscission bud’ cultivar ‘Tima Zhenzhu’ and ‘the non abscission bud’ cultivar ‘Dabanhong’ as materials. By integrating histology, widely targeted metabolomics, transcriptomics, and hormone quantification, we systematically analyzed the dynamic changes that occurred during key stages of bud programmed cell death (PCD).

**Results:**

During bud abscission, typical PCD features were observed, including degradation of the cytoskeleton, cell wall shrinkage, and disintegration of vacuoles, nuclei, and other organelles. Hormone dynamics further revealed that the levels of active cytokinins decreased, whereas auxin precursors, ABA, jasmonic acid, and ethylene accumulated markedly, jointly driving the PCD process. At the S25 and S30 stages, 705 and 4,731 differentially expressed genes (DEGs) were identified, along with 241 and 345 differentially accumulated metabolites (DAMs), respectively. These molecules were significantly enriched in pathways such as plant hormone signal transduction, secondary metabolite biosynthesis, sugar metabolism, and lipid metabolism. Based on multi omics data, this study predicts a co regulatory network for bud PCD, illustrating how ordered programmed cell death is achieved through suppression of growth signals, activation of autophagy and antioxidant systems, and reprogramming of secondary metabolic flux.

**Discussion:**

These findings not only revealed the intrinsic molecular landscape of bud abscission in ‘Tima Zhenzhu’, but also provided potential targets and strategic insights for future metabolic engineering or genetic improvement.

## 1 Introduction

**Figure 1:**
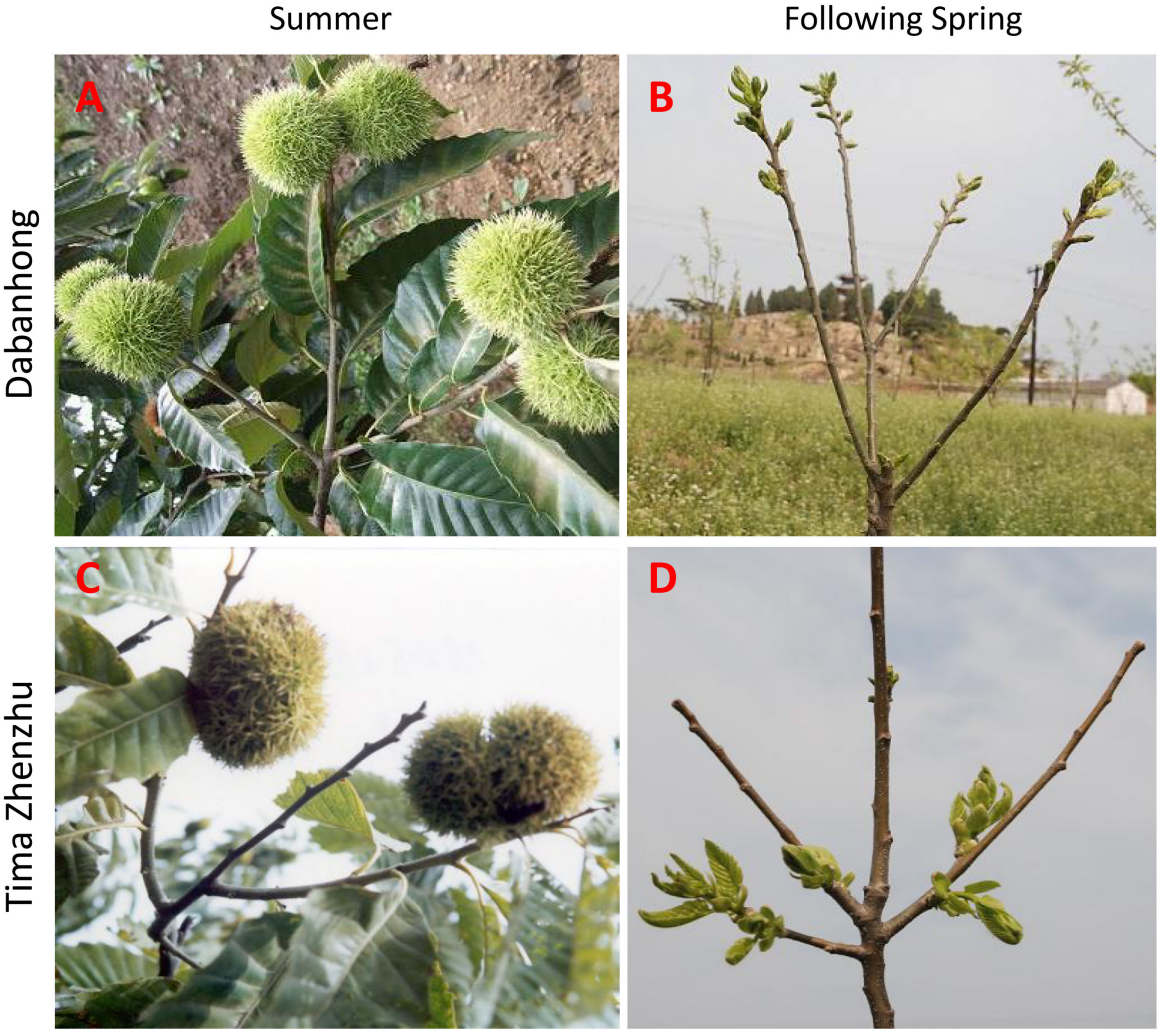
Comparison of shoot growth patterns between the ‘non abscission-bud’ cultivar ‘Dabanhong’ and the ‘abscission bud’ cultivar ‘Tima Zhenzhu’ in chestnut (*Castanea mollissima*). **(A, B)** ‘Dabanhong’: **(A)** shoot in summer showing apical bud retention; **(B)** shoot in the following spring, with the apical bud developing into a new fruiting branch. **(C, D)** ‘Tima Zhenzhu’: **(C)** shoot in summer showing apical bud abscission; **(D)** shoot in the following spring, with lower latent bud outgrowth forming a new branch.

Chestnut (*Castanea mollissima* BL.) is an important commercial nut-bearing tree species. Typically, the apical bud of each fruiting branch develops into the following year’s fruiting branch, a process that results in the majority of fruit being produced on the periphery of the crown (**Figures 1A, B**). The rapid expansion of the crown (averaging about 40 cm outward per year) not only increases labor costs for pruning management but also leads to a decline in tree productivity and lower land use efficiency over time. The development of intensive production requires both increased planting area and density, as well as the cultivation of dwarf varieties with compact crowns; these two aspects are complementary (Liu et al., 2004). The cultivar ‘Tima Zhenzhu’ is a spontaneous mutant first reported in 1979 (Liu et al., 2004). It possesses a unique ‘abscission bud’ (Tima) trait. Under normal growing conditions, the apical bud (replaceable bud) of each fruiting branch in ‘Tima Zhenzhu’ senesces and abscises in the summer. Growth then shifts to the lower latent buds, which develop into new branches for the following year (**Figures 1C, D**). As a result, the tree naturally forms a compact crown, making this cultivar an ideal natural model for studying dwarfing breeding and high-density planting. Previous research has established that the fundamental cause of this natural apical bud senescence and abscission trait in ‘Tima Zhenzhu’ chestnut is the occurrence of programmed cell death (PCD) in the bud cells at the apical shoot section (Wang et al., 2012). Therefore, in-depth analysis of the complex molecular regulatory network and metabolic reprogramming mechanisms underlying the bud PCD process in chestnut holds theoretical significance for the targeted regulation and utilization of this trait.

Programmed cell death (PCD) is an active and positive form of death that is genetically controlled and regulated by multiple factors. It is a fundamental biological process in plant development, stress response, and organ morphogenesis (Jones, 2001; Wang et al., 2021). PCD in plants is a highly orchestrated biological process involving multiple organelles—including mitochondria, chloroplasts, the endoplasmic reticulum, and vacuoles—and their dynamic interactions (Gutiérrez-Aguilar, 2020; Kirschner, 2023). Based on the nature of the inducing signals, plant PCD can be broadly classified into developmental PCD, which is triggered by internal genetic programs, and environmentally induced PCD, which arises in response to external stresses (Dauphinee et al., 2019; Bosch and Franklin-Tong, 2024). Developmental PCD occurs at predetermined times and locations to support normal plant function, such as during xylogenesis in poplar, as well as leaf and petal senescence (Kaya and Kose, 2019; Guo et al., 2021; Lone et al., 2025). The execution of PCD is regulated by a complex interplay of extracellular and intracellular signaling systems and involves cascades of gene activation and repression (Kabbage et al., 2017). The balance between PCD-inducing factors and survival signals that suppress PCD shapes the downstream response network, reflecting the physiological complexity of the process (Kabbage et al., 2017). Known signaling molecules implicated in plant PCD include Ca²^+^, reactive oxygen species (ROS), nitric oxide (NO), and phytohormones (Haghpanah et al., 2025; Zhang et al., 2015; Verma et al., 2019; Ren et al., 2021; Hussain et al., 2022; Ren et al., 2022). Numerous studies have shown that the PCD process involves multiple aspects such as rhythm, photosynthesis, nutrient remobilization, hypersensitive response, and autophagy (Yamada and Umehara, 2015; Zhang et al., 2015; Goto-Yamada et al., 2019; Verma et al., 2019; Valandro et al., 2020; Hussain et al., 2022; Feng et al., 2025).

To date, studies investigating programmed cell death (PCD) in buds of the chestnut cultivar ‘Tima Zhenzhu’ have remained limited. Wang et al. (2012) first defined the stages of PCD in this cultivar and characterized the corresponding ultrastructural changes in bud cells. Building upon this cytological foundation, our subsequent transcriptomic analysis (Guo et al., 2023) provided the first molecular insights into this process by comparing gene expression profiles of ‘Tima Zhenzhu’ buds at three key developmental stages: before, during, and following PCD. That study not only identified a substantial number of differentially expressed genes but also revealed that the plant hormone signal transduction pathway acts as a central hub in the regulatory network governing this process. Nevertheless, despite these advances, the specific metabolic reprogramming events, the dynamic interplay of multiple hormones, and the integrated regulatory networks that orchestrate this complex developmental process remain to be elucidated.

In this study, we employed *Castanea mollissima* ‘Tima Zhenzhu’ and ‘Dabanhong’ as representative materials and adopted an integrated multi-omics comparative biology approach to perform time-series sampling at the critical bud stages of PCD of ‘Tima Zhenzhu’. By coupling analyses of ultrastructural cellular features, quantitative plant hormone profiling, widely targeted metabolomics, and transcriptomics, this research aimed to construct a comprehensive spatiotemporal regulatory network model for chestnut bud PCD. This work not only provides overall insights into the intricate regulatory mechanisms underlying organ abscission but also potential PCD-related molecular targets for the precise modulation of tree architecture development via genetic or chemical strategies in *Castanea mollissima*.

## 2 Materials and methods

### 2.1 Plant plantation and phenotype collection

The plant materials used in this study, the chestnut cultivar ‘Tima Zhenzhu’ (with abscission buds) and the cultivar ‘Dabanhong’ (with non-abscission buds), were provided by the Changli Institute of Pomology, Hebei Academy of Agriculture and Forestry Sciences (**Figure 1**). The plants located at 118°51′ E, 39°53′ N in a region with a warm temperate semi-humid continental climate, an annual average temperature of 12.5 °C, and an annual precipitation of 650 mm (Wang et al., 2012; Guo et al., 2023). Both cultivars were grown under standard field management conditions in the institute’s germplasm repository, using gravelly sandy-loam soil with a planting spacing of 4m × 4 m. Nine healthy and normally growing ‘Tima Zhenzhu’ trees were selected, with each three as one biological replicate and twenty buds were collected from each tree.

The phenology of ‘Tima Zhenzhu’ and ‘Dabanhong’ was highly synchronized, with male-flower abscission occurring on June 20, 2024. For ‘Tima Zhenzhu’ (**Supplementary Figure 1**), buds of fruiting branch were collected at 20, 25, and 30 days after male-flower abscission in 2024, corresponding to the developmental stages S20 (pre-PCD), S25 (PCD initiated), and S30 (PCD largely complete). Upper buds from these stages were designated UBS20, UBS25, and UBS30, while lower buds collected at S25 and S30 were labeled LBS25 and LBS30, respectively. For comparison, upper buds of the non-abscission cultivar ‘Dabanhong’ were sampled at the same time points and labeled NYS20, NYS25, and NYS30 (**Supplementary Figure 1**). Immediately after collection, bud scales were removed, and all samples were frozen in liquid nitrogen quickly. Each pooled sample (by stage and position) was divided into three aliquots for hormone profiling, RNA-Seq, and qRT-PCR analysis, and stored at -80°C until use.

### 2.2 Ultrastructural observation of buds at different stages

Preparation and observation of ultrathin sections followed the protocol described by Yang et al (Yang et al., 2022b). The base tissue of buds was excised and trimmed into 0.5 cm × 0.5 cm pieces, then immersed in 2.5% glutaraldehyde solution. After vacuum infiltration, samples were stored at 4 °C. Dehydration was performed through a graded ethanol series (30%, 50%, 70%, 80%, 90%), 10 min per step, followed by three 30-min treatments in absolute ethanol. Infiltration was carried out sequentially with mixtures of ethanol and Spurr’s resin at ratios of 3:1, 1:1, and 1:3 for 2 h, 5 h, and 12 h, respectively. Tissues were then embedded in capsules and polymerized at 55 °C for 48 h. Ultrathin sections (60–70 nm) were cut, stained with 2% uranyl acetate for 30 min, washed five times with ddH_2_O, air-dried, and examined under a transmission electron microscope to observe cellular ultrastructure.

### 2.3 Detection of phytohormone abundance and metabolome

Phytohormone levels and wide-target metabolomic profiles were analyzed using an AB Sciex QTRAP 6500 LC-MS/MS platform (MetWare, http://www.metware.cn/). Fresh bud samples were collected, immediately frozen in liquid nitrogen, and ground into powder (30 Hz, 1 min) before storage at −80 °C until use. For extraction, 50 mg of powdered sample was placed into a 2 mL microtube, flash-frozen in liquid nitrogen, and dissolved in 1 mL of methanol/water/formic acid (15:4:1, V/V/V). Then, 10 μL of an internal standard mixture (100 ng/mL) was added for quantification. After vortexing for 10 min, the mixture was centrifuged at 12,000 r/min for 5 min at 4 °C. The supernatant was transferred to a new microtube, evaporated to dryness, reconstituted in 100 μL of 80% methanol (V/V), and filtered through a 0.22 μm membrane before LC-MS/MS analysis (Qin et al., 2025).

### 2.4 Analyses of phytohormone abundance and metabolome

Significantly regulated metabolites between groups were determined by absolute Log2FC (fold change) and *P*-values. Identified metabolites were annotated using the KEGG compound database (http://www.kegg.jp/kegg/compound/). Annotated metabolites were then mapped to the KEGG Pathway database (http://www.kegg.jp/kegg/pathway.html), and their significance was determined by the hypergeometric test’s p-values.

### 2.5 LC-MS/MS platform and settings

Sample extracts were analyzed on a UPLC-ESI-MS/MS system, including an ExionLC™ AD UPLC (https://sciex.com.cn/) coupled to a tandem mass spectrometer (https://sciex.com.cn/). The UPLC separation used an Agilent SB-C18 column (1.8 µm, 2.1 × 100 mm) maintained at 40 °C. The mobile phase consisted of solvent A (pure water with 0.1% formic acid) and solvent B (acetonitrile with 0.1% formic acid). The gradient program started at 95% A and 5% B, linearly shifted to 5% A and 95% B over 9 min, held for 1 min, returned to 95% A and 5% B in 1.1 min, and re-equilibrated for 2.9 min. The flow rate was 0.35 mL/min, and the injection volume was 2 μL. Column effluent was directed to an ESI-triple quadrupole-linear ion trap (QTRAP) mass spectrometer.

ESI source conditions were as follows: source temperature, 500 °C; ion spray voltage, 5500 V (positive) or −4500 V (negative); ion source gas I (GSI), gas II (GSII), and curtain gas (CUR) set to 50, 60, and 25 psi, respectively; collision-activated dissociation (CAD), high. MRM experiments were conducted using QQQ scans with nitrogen as the collision gas (medium setting). Declustering potential (DP) and collision energy (CE) were optimized for each MRM transition. During analysis, specific MRM transitions were monitored corresponding to metabolites eluting in each time window.

### 2.6 RNA isolation and RNA-seq analyses

The construction of cDNA libraries and subsequent sequencing were conducted by Metware Biotechnology Co., Ltd (Wuhan, China). Raw sequencing reads were processed with fastp for quality control, which included the removal of adapter sequences and low-quality reads (Chen et al., 2018). Cleaned reads were then mapped to the Chinese Chestnut (*Castanea* mollissima) reference genome employing Hisat 2.0 under default parameters (Wang et al., 2020; Hufford et al., 2021). Novel gene identification was carried out using Stringtie software, while gene expression levels were quantified with FeatureCounts software. Finally, FPKM (fragments per kilobase of exon per million mapped fragments) values were computed for subsequent analyses.

Differentially expressed genes (DEGs) between groups were identified using DESeq2, with *padj* values (Benjamini–Hochberg correction) and |log_2_ foldchange| serving as significance thresholds. Functional enrichment was subsequently assessed via hypergeometric tests: KEGG pathway enrichment was analyzed at the pathway level, and Gene Ontology (GO) enrichment was evaluated by GO term.

### 2.7 Weighted gene co-expression network analysis

Weighted gene co-expression network analysis (WGCNA) was applied to explore coordinated expression patterns (Langfelder and Horvath, 2008). The weighted coefficient *β* was determined using the pickSoftThreshold function. A value of soft thresholding was selected for network construction. Based on this, an adjacency matrix was computed and converted into a topological overlap matrix. Modules were then identified using the dynamic tree cut algorithm with default parameters. To identify modules associated with specific traits, a grouping matrix derived from the 24 samples was used as trait data for module–trait relationship analysis. Finally, the co-expression network was generated in STRING v12 (https://string-db.org/) and visualized using Cytoscape v3.5.1. The extent of PCD in each sample was quantified and used to construct the sample-trait matrix. Apical buds of ‘Tima Zhenzhu’ at stage S25 and S30 were assigned scores of 0.5 and 1, respectively, reflecting intermediate and advanced PCD progression, while samples without observable PCD were scored as 0. In addition, the abundance of strongly correlated hormone compounds was included as traits in the WGCNA.

### 2.8 Quantitative real-time PCR analysis

Total RNA extracted from different tissues of the two cultivars was reverse-transcribed into cDNA using the HiScript II Q RT SuperMix for qPCR (Yisheng Biotechnology, China). The resulting cDNA served as the template for quantitative real-time PCR (qRT-PCR). All reactions were performed on a CFX96 real-time PCR system (Bio-Rad) using the Hieff ^®^ qPCR SYBR Green Master Mix. *EF1-α* was used as the internal reference gene (Guo-Song et al., 2019). Each 10 μL reaction contained 100 ng of cDNA, 0.8 μmol/L of gene-specific primers, and 5 μL of the qPCR Mix. The thermal cycling protocol was as follows: initial denaturation at 95 °C for 1 min, followed by 40 cycles of 95 °C for 10 s and 55 °C for 5 s. A melting curve analysis was subsequently performed (95 °C for 10 s, 55 °C for 10 s, and 72 °C for 15 s). Relative gene expression levels were calculated using the 2^−ΔΔCT^ method. with *EF1-α* serving as the internal reference gene and UBS20 as the calibrator sample (the referent treatment). Sequences of the primers used are listed in **Supplementary Table 1**.

## 3 Results

### 3.1 Ultrastructural changes in buds across developmental stages

**Figure 2:**
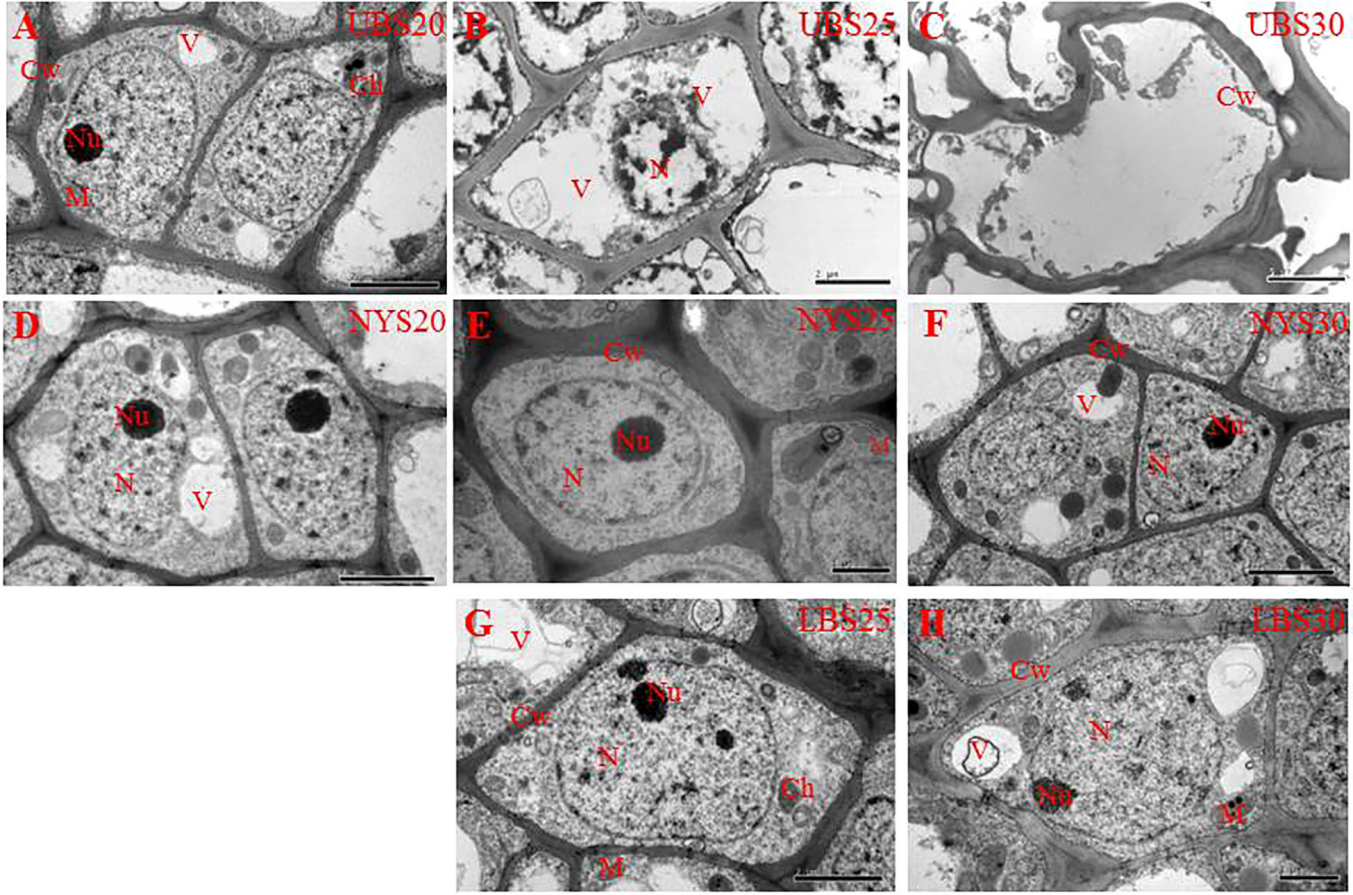
Transmission electron micrographs of cell ultrastructure during bud senescence. **(A)** UBS20; **(B)** the UBS25; **(C)** UBS30; **(D)** NYS20; **(E)** NYS25; **(F)** NYS30; **(G)** LBS25; **(H)** LBS30. UB, upper buds of ‘Tima Zhenzhu’; LB, lower buds of ‘Tima Zhenzhu’; NY, upper buds of ‘Dabanhong’. S20, S25, and S30 were the stages 20, 25, and 30 days after male flower abscission, respectively. Bars = 2 μm. Ch, chloroplast; Cw, cell wall; M, mitochondrion; N, nucleus; Nu, nucleolus; Pm, plasmalemma; V, vacuole.

During bud development, marked differences in cellular structure emerged among the upper buds of ‘Tima Zhenzhu’, its lower buds, and the upper buds of ‘Dabanhong’ (**Figure 2**). At the S20 stage, all buds exhibited typical cellular architecture, featuring vacuolated cytoplasm, clearly visible chloroplasts and mitochondria, a dense cytoplasmic matrix, intact cell and organelle membranes, and round nuclei with evenly distributed chromatin. No PCD-related changes were detected at this stage. By the S25 stage, the upper buds of ‘Tima Zhenzhu’ showed pronounced ultrastructural alterations indicative of active PCD (**Figure 2B**). Vacuoles were notably enlarged with ruptured tonoplasts, cell walls appeared thickened and partially fractured, and mitochondria and chloroplasts were no longer discernible. Nuclei were visibly shrunken, with condensed chromatin and absent nucleoli. At the S30 stage, cells in the upper buds of ‘Tima Zhenzhu’ had undergone cell death (**Figure 2C**). The cytoskeleton was severely degraded or deformed, cell walls were shrunken, and vacuoles, nuclei, and other organelles were largely fragmented and disintegrated. PCD was essentially complete at this stage, and buds detached readily with slight physical disturbance. In contrast, the upper buds of ‘Dabanhong’ at both S25 and S30, as well as the lower buds of ‘Tima Zhenzhu’ (**Figures 2D–H**), retained the typical cellular morphology observed at S20, showing no signs of PCD progression.

### 3.2 Phytohormone compound accumulation changes

**Figure 3:**
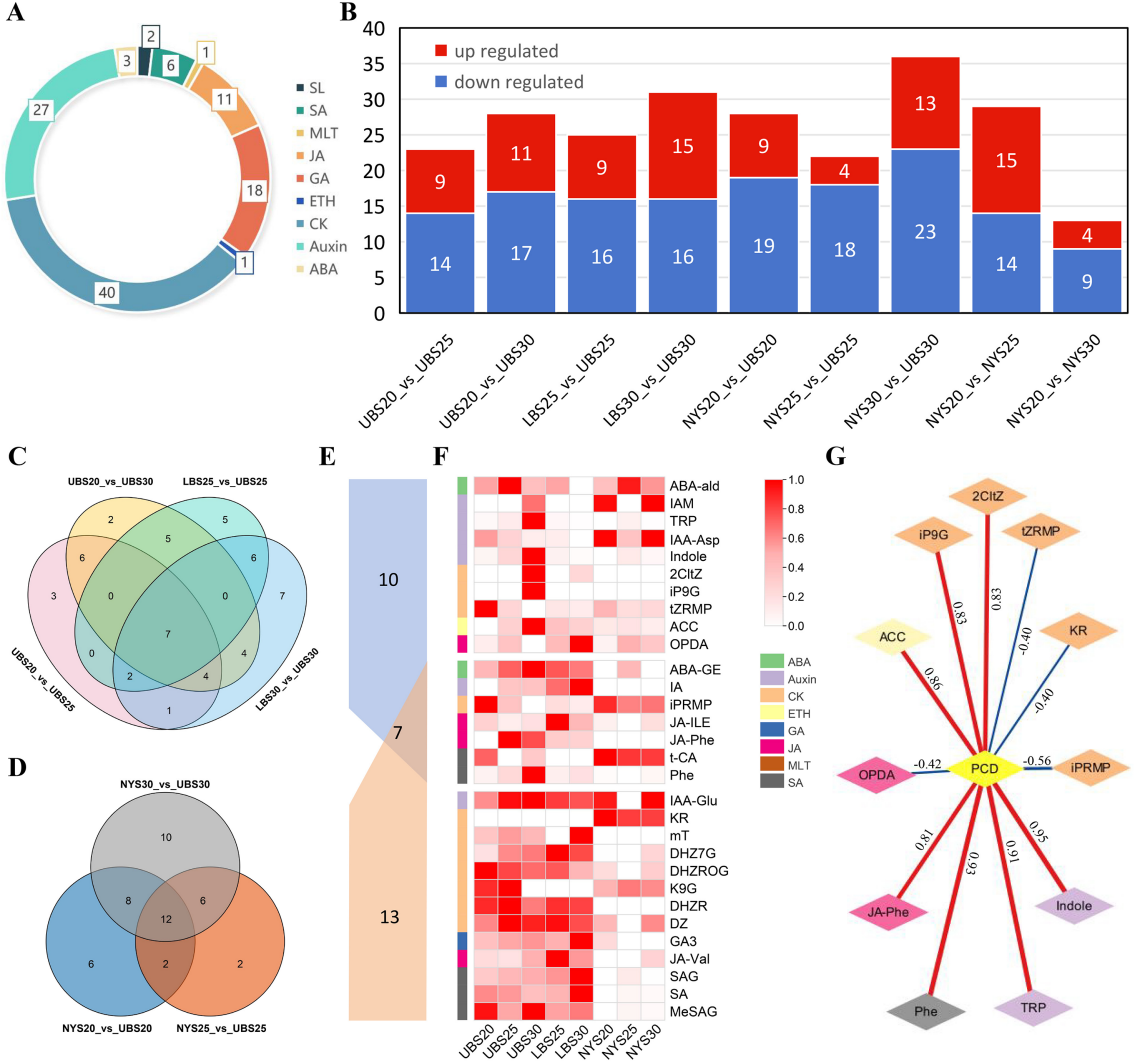
Analysis of phytohormone accumulation patterns in *Castanea mollissima* buds. **(A)** Categories and numbers of detected phytohormone compounds. Colors represent different hormone classes. **(B)** Numbers of differentially accumulated hormones (DAHs) identified in pairwise comparisons of different bud groups. Venn diagrams showing differentially expressed genes (DEGs) identified among **(D)** upper and lower buds of ‘Tima Zhenzhu’ and among **(C)** ‘Dabanhong’ and ‘Tima Zhenzhu’. **(E)** Venn diagrams of the DAHs between **(C, D)**. **(F)** Heatmap displaying the abundance of hormone compounds shared across multiple comparisons. Abundance values were normalized. Darker shades indicate higher abundance levels. CK, cytokinins; ETH, ethylene; GA, gibberellins; JA, jasmonates; SA, salicylic acids; ABA, abscisic acid; SL, strigolactones; MLT, melatonin. **(G)** Correlation network between PCD progression and phytohormone compounds. Pearson correlation coefficients are shown on the connections; red edges indicate positive correlations, blue edges indicate negative correlations.

Given the proposed importance of phytohormones in bud abscission of ‘Tima Zhenzhu’, we quantified multiple hormone classes across three developmental stages (S20, S25, S30). The analysis covered auxins (27 compounds), cytokinins (40), ethylene (1), gibberellins (18), jasmonates (11), salicylic acids (6), abscisic acid (3), strigolactones (2), and melatonin (1) (**Figure 3A**; **Supplementary Table 2**). Differentially accumulated hormones (DAHs) were identified in the following pairwise comparisons: UBS20 vs. UBS25 (23 DAHs), UBS20 vs. UBS30 (28), LBS25 vs. UBS25 (25), LBS30 vs. UBS30 (31), NYS20 vs. UBS20 (28), NYS25 vs. UBS25 (22), NYS30 vs. UBS30 (36), NYS20 vs. NYS25 (29), and NYS20 vs. NYS30 (13) (**Figure 3B**).

Within ‘Tima Zhenzhu’, comparisons between upper and lower buds identified seven shared DAHs (IAA-Asp, tZRMP, iPRMP, Indole, JA-Phe, IA, and Phe) (**Figure 3C**). Four compounds (ABA-GE, ACC, t-CA, and OPDA) were shared across the UBS20 vs. UBS25, UBS20 vs. UBS30, and LBS30 vs. UBS30 comparisons. Two (ABA-ald and JA-ILE) were shared across UBS20 vs. UBS25, LBS25 vs. UBS25, and LBS30 vs. UBS30; and four (2CltZ, iP9G, TRP, and IAM) were specific to UBS20 vs. UBS30 and LBS30 vs. UBS30.

Between cultivars ‘Tima Zhenzhu’ and ‘Dabanhong’, 12 DAHs were consistently detected, including ABA-GE, DHZ7G, mT, KR, MeSAG, SA, GA3, K9G, SAG, JA-Val, DHZR, and DHZROG (**Figure 3D**). Six compounds (t-CA, iPRMP, DZ, JA-Phe, IA, and Phe) overlapped in both NYS25 vs. UBS25 and NYS30 vs. UBS30, while two (IAA-Glu and JA-ILE) were common to NYS20 vs. UBS20 and NYS25 vs. UBS25.

Notably, seven core DAHs (ABA-GE, IA, iPRMP, JA-Phe, JA-ILE, t-CA, and Ph) were shared between intra-cultivar (‘Tima Zhenzhu’) and inter-cultivar (‘Tima Zhenzhu’ vs. ‘Dabanhong’) comparisons (**Figure 3E**). Most showed significant differences between PCD and non-PCD buds (**Figure 3F**). ABA-ald, ABA-GE, IAM, TRP, Indole, 2CltZ, iP9G, ACC, JA-Phe, and Phe peaked in UBS25 or UBS30, whereas tZRMP, iPRMP, and K9G reached their lowest levels in these stages. We further performed Pearson correlation analysis to assess the relationships between PCD progression and the abundance of DAHs. The correlation coefficients ranged from -0.56 to 0.94 across the 30 DAHs examined (**Figure 3G**; **Supplementary Figure 2**). Notably, PCD progress showed strong positive correlations with Indole (*r* = 0.95), Phe (*r* = 0.93), TRP (*r* = 0.91), ACC (*r* = 0.86), iP9G (*r* = 0.83), 2CltZ (*r* = 0.83), JA-Phe (*r* = 0.81), and ABA-GE (*r* = 0.65). Moderate negative correlations were observed between PCD and iPRMP (*r* = -0.56), OPDA (*r* = -0.42), KR (*r* = -0.40), tZRMP (*r* = -0.40), IAA-Asp (*r* = -0.38), and t-CA (r = -0.31). These findings suggest potential biological relevance of these hormone compounds in the PCD process.

### 3.3 Metabolomic changes in buds across developmental stages

**Figure 4:**
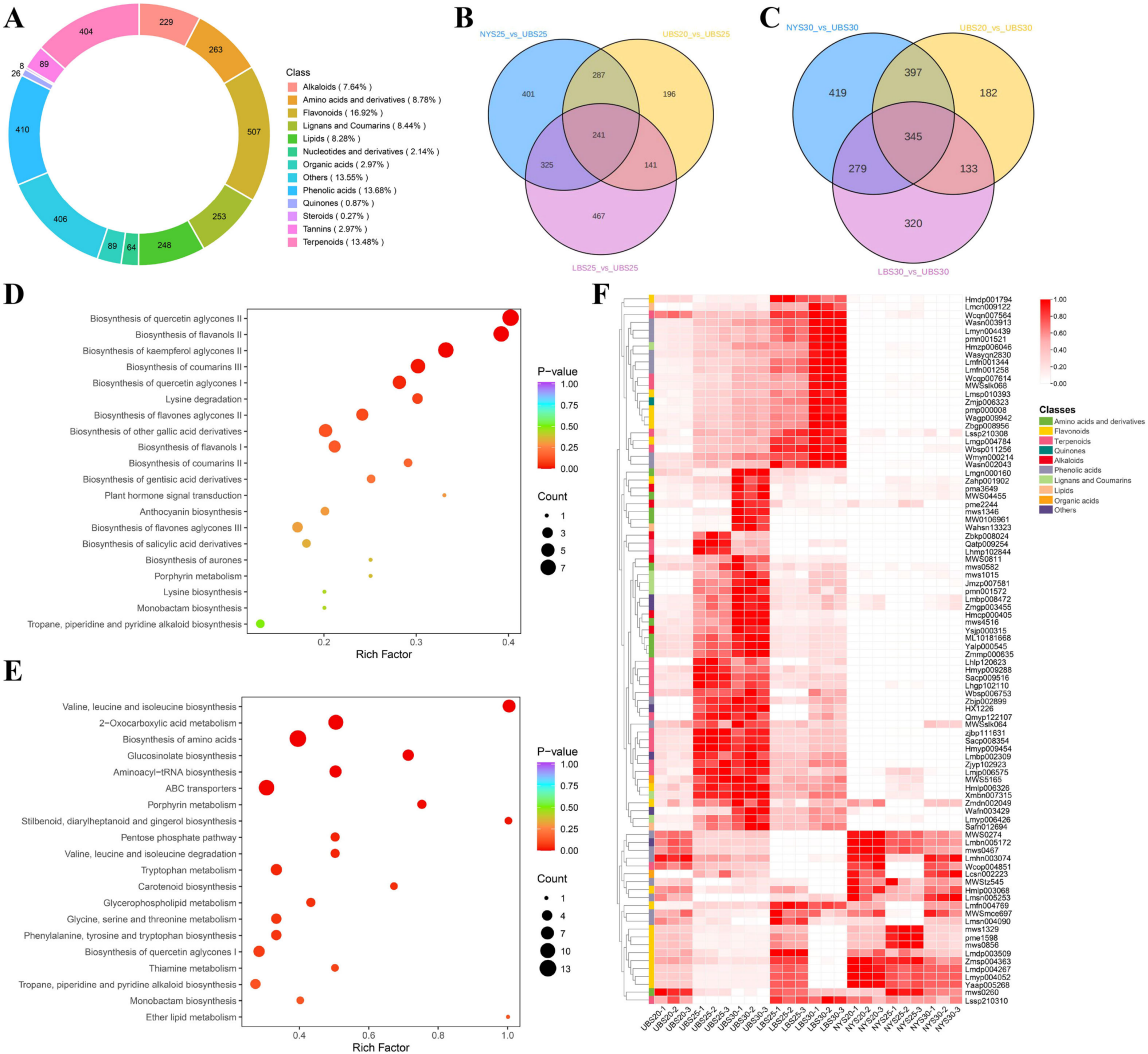
Metabolomic profiling of 24 bud samples. **(A)** Overall classification of detected metabolites. **(B)** Venn diagram showing the overlap of differentially accumulated metabolites (DAMs) among the comparisons UBS20 vs. UBS25, LBS25 vs. UBS25, and NYS25 vs. UBS25. **(C)** Venn diagram showing the overlap of DAMs among UBS20 vs. UBS30, LBS30 vs. UBS30, and NYS30 vs. UBS30. **(D)** KEGG pathway enrichment analysis of DAMs common to UBS20 vs. UBS25, LBS25 vs. UBS25, and NYS25 vs. UBS25. Larger bubbles indicate a greater number of annotated genes. **(E)** KEGG pathway enrichment analysis of DAMs common to UBS20 vs. UBS30, LBS30 vs. UBS30, and NYS30 vs. UBS30. **(F)** Heatmap showing abundance patterns of the 90 DAMs consistently detected across comparisons. Metabolite abundance values were normalized by Z-score. Colors denote metabolite classes.

To elucidate metabolic reprogramming during PCD, we carried out a widely targeted metabolomics analysis. Technical reproducibility was validated by tight clustering in PCA and strong inter-QC correlations (**Supplementary Figure 3**). These metabolites were classified into 13 categories, including flavonoids (507, 16.9%), phenolic acids (410, 13.7%), terpenoids (404, 13.5%), amino acids and derivatives (263, 8.8%), lignans and coumarins (253, 8.4%), lipids (248, 8.3%), alkaloids (229, 7.6%), tannins (89, 3.0%), organic acids (89, 3.0%), nucleotides and derivatives (64, 2.1%), quinones (26, 0.9%), steroids (8, 0.3%), and others (406, 13.6%)(**Figure 4A**; **Supplementary Table 3**). Differentially abundant metabolites (DAMs) were identified in nine pairwise comparisons (**Supplementary Figure 4**), UBS20 vs. UBS25 (865 DAMs), UBS20 vs. UBS30 (1,057), LBS25 vs. UBS25 (1,174), LBS30 vs. UBS30 (1,077), NYS20 vs. UBS20 (1,178), NYS25 vs. UBS25 (1,254), NYS30 vs. UBS30 (1,440), NYS20 vs. NYS25 (1,065), and NYS20 vs. NYS30 (362).

In the S25-stage comparisons (UBS20 vs. UBS25, LBS25 vs. UBS25, and NYS25 vs. UBS25), 241 DAMs were commonly identified (**Figure 4B**). These metabolites were significantly enriched in flavonoid-biosynthesis pathways (**Figure 4D**; **Supplementary Table 4**), such as the biosynthesis of quercetin aglycones II (MetMap116, *p* = 0.0006), flavanols II (MetMap141, *p* = 0.0017), kaempferol aglycones II (MetMap114, *p* = 0.0048), coumarins III (MetMap129, *p* = 0.016), and quercetin aglycones I (MetMap115, *p* = 0.038).

At the S30 stage (UBS20 vs. UBS30, LBS30 vs. UBS30, and NYS30 vs. UBS30), 345 common DAMs were detected (**Figure 4C**). These showed significant enrichment in multiple KEGG pathways (**Figure 4E**), including biosynthesis of secondary metabolites (ko01110, *p* = 5.39E-15), plant hormone signal transduction (ko04075, *p* = 3.67E-09), glucosinolate biosynthesis (ko00966, *p* = 5.9E-04), aminoacyl-tRNA biosynthesis (ko00970, *p* = 0.002), ABC transporters (ko02010, *p* = 0.005), porphyrin metabolism (ko00860, *p* = 0.008), stilbenoid, diarylheptanoid and gingerol biosynthesis (ko00945, *p* = 0.017), valine, leucine and isoleucine degradation (ko00280, *p* = 0.032), tryptophan metabolism (ko00380, *p* = 0.035), carotenoid biosynthesis (ko00906, *p* = 0.046), and glycerophospholipid metabolism (ko00564, *p* = 0.05).

Furthermore, 90 DAMs were consistently significant across all comparisons of UBS30 against other developmental stages (**Figure 4F**). Among these, down-regulation of several amino acids (e.g., L-arginine) and most flavonols corresponded to reduced growth and attenuated primary metabolism. In contrast, a suite of alkaloids, free fatty acids, organic-acid glycosides, lignans, coumarins, and the majority of terpenoids (85%) were markedly up-regulated. These coordinated shifts reflected a metabolic reprogramming in which growth- and maintenance-related pathways were suppressed, whereas defense- and signaling-associated pathways were strongly activated.

### 3.4 Gene expression changes in buds across developmental stages

To capture transcriptional dynamics during bud PCD, RNA-seq was performed on all 24 samples. Sequencing libraries yielded high-quality reads, with Q20 and Q30 scores exceeding 98.0% and 94.1%, respectively (**Supplementary Table 5**). Clean reads per sample ranged from 45.8 to 63.6 million, and over 88.4% were successfully mapped to the reference genome (**Supplementary Table 5**). Principal component analysis (PCA) showed tight clustering within developmental stages and high reproducibility across biological replicates (**Supplementary Figure 5**), supporting the reliability of library preparation and sequencing. Alignment against the reference genome identified 4,493 novel transcripts, all of which were subsequently annotated (**Supplementary Table 6**).

**Figure 5:**
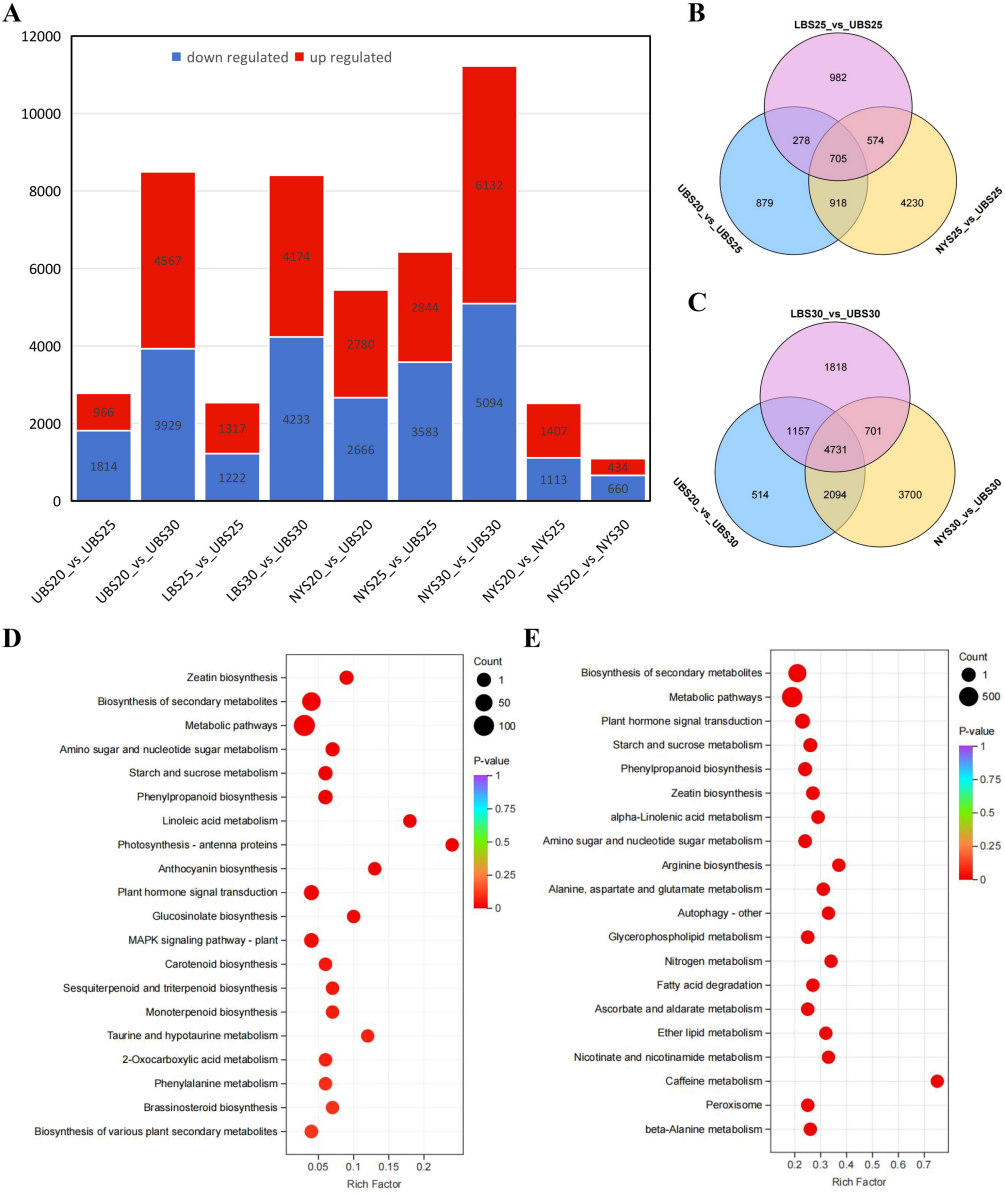
RNA-seq analyses of 24 bud samples. **(A)** The number of differentially expressed genes (DEGs) among different groups. **(B)** Venn diagram showing the overlap of DEGs among the comparisons UBS20 vs. UBS25, LBS25 vs. UBS25, and NYS25 vs. UBS25. **(C)** Venn diagram showing the overlap of DEGs among UBS20 vs. UBS30, LBS30 vs. UBS30, and NYS30 vs. UBS30. **(D)** KEGG pathway enrichment analysis of DEGs common to UBS20 vs. UBS25, LBS25 vs. UBS25, and NYS25 vs. UBS25. Larger bubbles indicate a greater number of annotated genes. **(E)** KEGG pathway enrichment analysis of DEGs common to UBS20 vs. UBS30, LBS30 vs. UBS30, and NYS30 vs. UBS30.

Differential expression analysis revealed 2,780, 2,539, and 6,427 DEGs in the comparisons UBS20 vs. UBS25, LBS25 vs. UBS25, and NYS25 vs. UBS25, respectively, with a balanced distribution of up- and down-regulated genes (**Figure 5A**). Among these, 705 DEGs were common to all three S25-stage comparisons (**Figure 5B**). In comparisons at S30 (UBS20 vs. UBS30, LBS30 vs. UBS30, and NYS30 vs. UBS30), 8,496, 8,407, and 11,226 DEGs were identified, respectively, with 4,731 DEGs shared across all three comparisons (**Figure 5C**).

KEGG enrichment analysis of the 705 shared DEGs at S25 showed significant enrichment in pathways related to plant hormone signal transduction (ko04075, ko00908), secondary metabolite biosynthesis (ko01110), general metabolism (ko01100), amino sugar and nucleotide sugar metabolism (ko00520), starch and sucrose metabolism (ko00500), phenylpropanoid biosynthesis (ko00940), linoleic acid metabolism (ko00591), photosynthesis (ko00196), anthocyanin biosynthesis (ko00942), glucosinolate biosynthesis (ko00966), and MAPK signaling (ko04016) (**Figure 5D**; **Supplementary Table 7**). The 4,731 shared DEGs at S30 were enriched in plant hormone signal transduction (ko04075), starch and sucrose metabolism (ko00500), phenylpropanoid biosynthesis (ko00940), zeatin biosynthesis (ko00908), α-linolenic acid metabolism, autophagy (ko04136), circadian rhythm (ko04712), photosynthesis (ko00196), carotenoid biosynthesis (ko00906), and fatty acid metabolism (ko00071, ko00592, and ko00591) (**Figure 5E**; **Supplementary Table 7**).

DEGs shared between the S25 and S30 stages were significantly enriched across multiple KEGG pathways, including zeatin biosynthesis (ko00908), secondary metabolite biosynthesis (ko01110), general metabolic pathways (ko01100), amino sugar and nucleotide sugar metabolism (ko00520), starch and sucrose metabolism (ko00500), phenylpropanoid biosynthesis (ko00940), linoleic acid metabolism (ko00591), photosynthesis—antenna proteins (ko00196), and plant hormone signal transduction (ko04075). Gene Ontology (GO) enrichment analysis yielded complementary results, with significant over-representation of the related biological processes and molecular functions (**Supplementary Figure 6**; **Supplementary Table 8**). Taken together, these transcriptomic patterns align with the metabolomic profiles, demonstrating that the PCD process in ‘Tima Zhenzhu’ buds entails tightly coordinated changes in both gene expression and metabolite accumulation.

### 3.5 Construction of gene-weighted co-expression network modules

**Figure 6:**
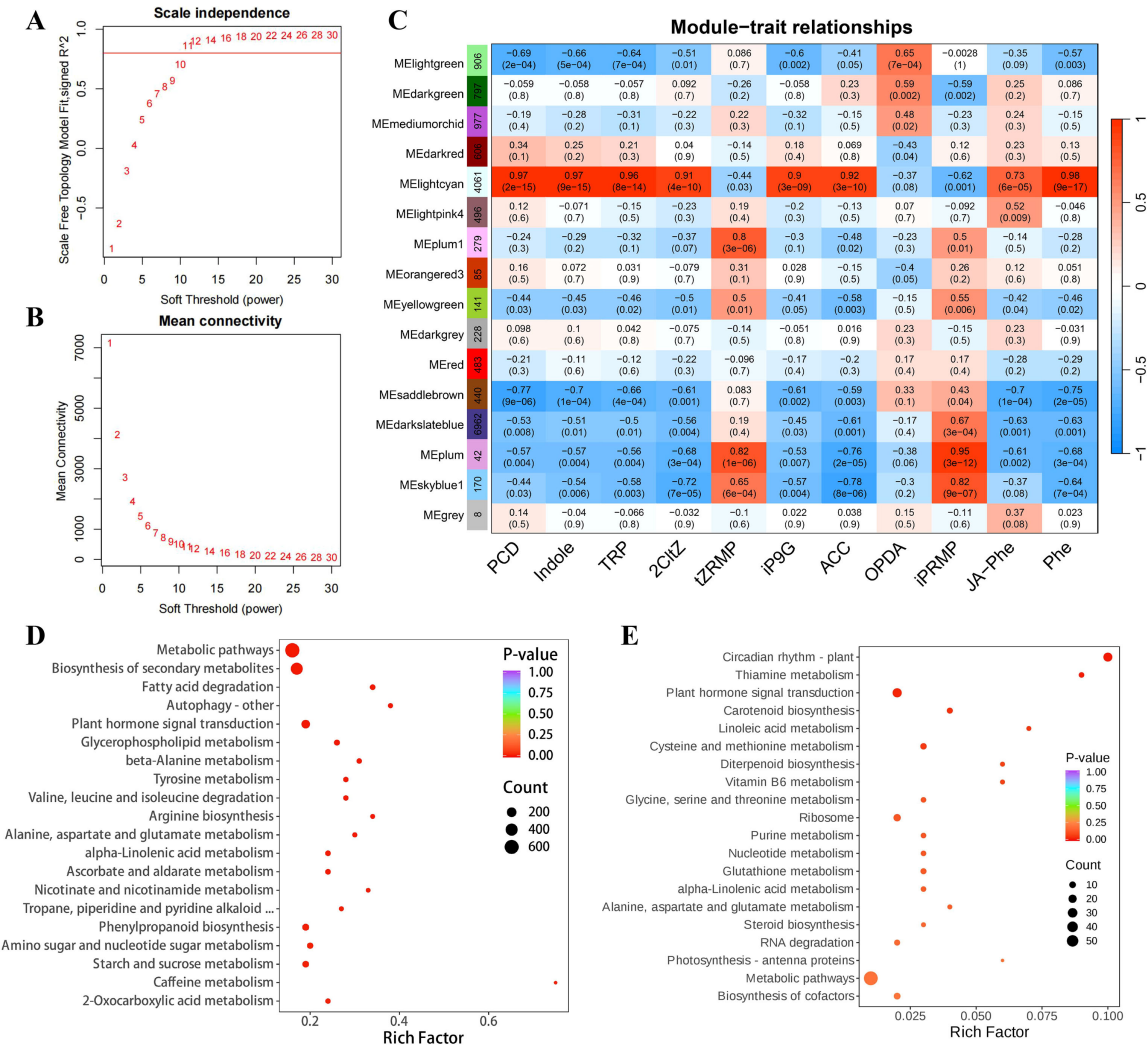
Construction and analysis of the weighted gene co-expression network. **(A)** Analysis of network topology under different soft-thresholding power *β*. **(B)** Mean connectivity change as a function of the soft-thresholding power *β*. **(C)** The correlation between modules and the PCD process. KEGG pathway enrichment analyses of genes in the **(D)** lightcyan and **(E)** saddlebrown modules.

A weighted gene co-expression network was constructed using DEGs from all 24 samples. The soft-thresholding power was set to *β* = 10, at which the scale-free topology fit index reached *R²* = 0.9 (**Figures 6A, B**), satisfying the criterion for building a scale-free network. Using the dynamic tree-cut algorithm, genes were clustered and partitioned into 16 distinct modules, ranging in size from 8 to 6,962 genes (**Figure 6C**; **Supplementary Figure 7**). The largest modules were darkslateblue (6,962 genes) and lightcyan (4,061 genes). Module-trait correlation analysis revealed varying degrees of association between co-expression modules and PCD-related traits across samples, with absolute correlation coefficients (*r*) ranging from 0.01 to 0.98 (**Figure 6C**). Notably, the lightcyan module showed strong positive correlations with PCD, Indole, TRP, 2CltZ, iP9G, ACC, and Phe (*r* ≥ 0.90), while being negatively correlated with tZRMP, OPDA, and iPRMP (|*r* |≥ 0.37)(**Figure 6C**). Most genes in this module exhibited peak expression during the UBS25 and UBS30 stages (**Supplementary Figure 8**). In contrast, the saddlebrown module displayed significant negative correlations with PCD, Indole, TRP, 2CltZ, iP9G, ACC, and Phe (| *r* |≥ 0.59), and the majority of its genes were expressed at the lowest levels in UBS30 (**Figure 6C**; **Supplementary Figure 8**). These two modules are likely key co-expression modules significantly associated with the PCD process in chestnut buds.

KEGG enrichment analyses further highlighted distinct functional profiles for these two key modules. Genes within the lightcyan module were significantly enriched in metabolic pathways (ko01100, *p* = 8.66E-10), biosynthesis of secondary metabolites (ko01110, *p* = 5.84E-6), fatty acid degradation (ko00071, *p* = 7.11E-6), autophagy (ko04136, *p* = 7.35E-5), plant hormone signal transduction (ko04075, *p* = 8.02E-5), glycerophospholipid metabolism (ko00564, *p* = 1.93E-4), and beta-alanine metabolism (ko00410, *p* = 2.05E-4) (**Figure 6D**; **Supplementary Table 9**). The saddlebrown module was primarily enriched in circadian rhythm—plant (ko04712, *p* = 4.99E-10), thiamine metabolism (ko00730, *p* = 7.23E-4), plant hormone signal transduction (ko04075, *p* = 0.016), carotenoid biosynthesis (ko00906, *p* = 0.035), linoleic acid metabolism (ko00591, *p* = 0.045), and cysteine and methionine metabolism (ko00270, *p* = 0.049) (**Figure 6E**; **Supplementary Table 9**).

**Figure 7:**
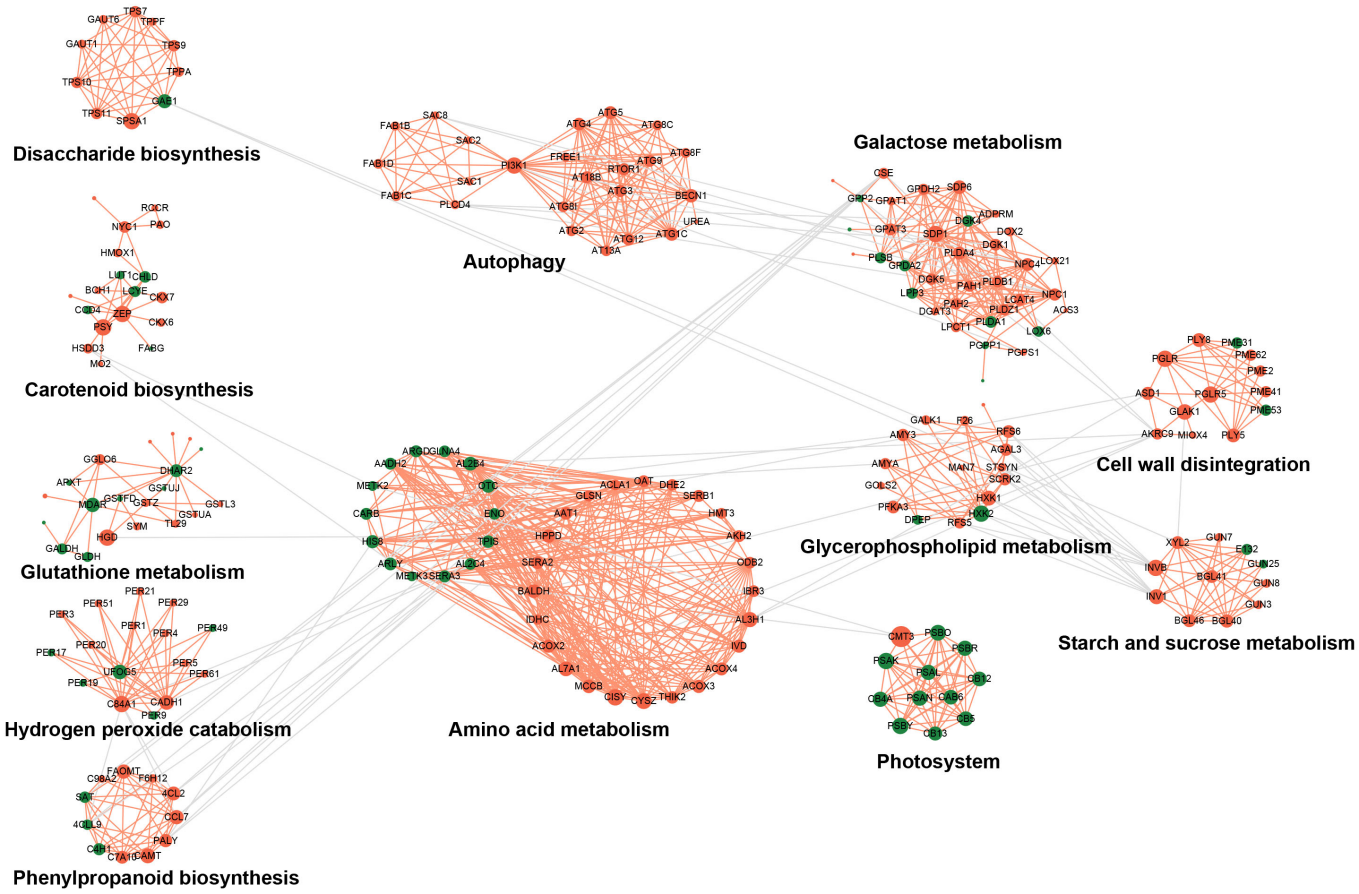
Protein association network for buds during the PCD progresses. The interaction network is predicted by STRING v12.0 online. Red and green nodes denote up- and down-regulated genes during the development of buds. The orange line indicates the interaction within a network, while the gray line represents the interaction between different networks.

To further elucidate the regulatory network underlying bud PCD, protein–protein interaction (PPI) networks were constructed using genes from the lightcyan and saddlebrown modules. These networks were subsequently partitioned into distinct gene clusters via the Markov Cluster Algorithm (MCL) (**Supplementary Table 11**). Functional annotation revealed that the resulting clusters were primarily associated with key physiological processes during PCD, including autophagy, redox homeostasis, photosystem function, amino acid metabolism, cell wall metabolism, sugar metabolism, and phenylpropanoid biosynthesis (**Figure 7**). Within the redox homeostasis network, phenylpropanoid biosynthesis, glutathione metabolism, and hydrogen peroxide catabolism emerged as central networks. Key genes in the phenylpropanoid pathway exhibited shapely upregulation, including *PALY*, CoA ligase family (*4CL2* and *CCL7*), and glutathione S-transferase family genes (*GSTs*). Peroxidase genes *PER1/3/4/5/61* were induced, whereas *PER9/17/19/49/72* were downregulated. In contrast, photosystem-associated genes were uniformly repressed, while *CMT3*, encoding a DNA methyltransferase, was markedly upregulated. The amino acid metabolic network was reprogrammed, characterized by the suppression of nitrogen assimilation genes (*GLNA4*, *CARB*, *OTC*, and *ARGD*) and the genes involved in carbon skeleton provision—including *CISY*, *CYSZ*, *IDHC*, and *ACLA1*—were predominantly upregulated. Cell wall metabolism was characterized primarily by the activation of pectin catabolic pathways, with highly connected hub genes including *PMEs*, *PGLRs*, and *PLY8/5*. The sugar metabolism network, encompassing cellulose, hemicellulose, glucan, and sucrose metabolism, involved key genes such as *INV1*, *PME62*, *PGs*, *XYL2*, and *GUN3/7/8/25*. Notably, we identified 47 autophagy-related genes in the chestnut genome by BLAST against Arabidopsis homologs (**Supplementary Table 10**). Among these, 17 autophagy genes were assigned to the lightcyan module (**Supplementary Table 10**), further supporting the involvement of this module in PCD of the upper buds in “Tima Zhenzhu”.

### 3.6 Regulatory Network of Differential Metabolism in PCD

**Figure 8:**
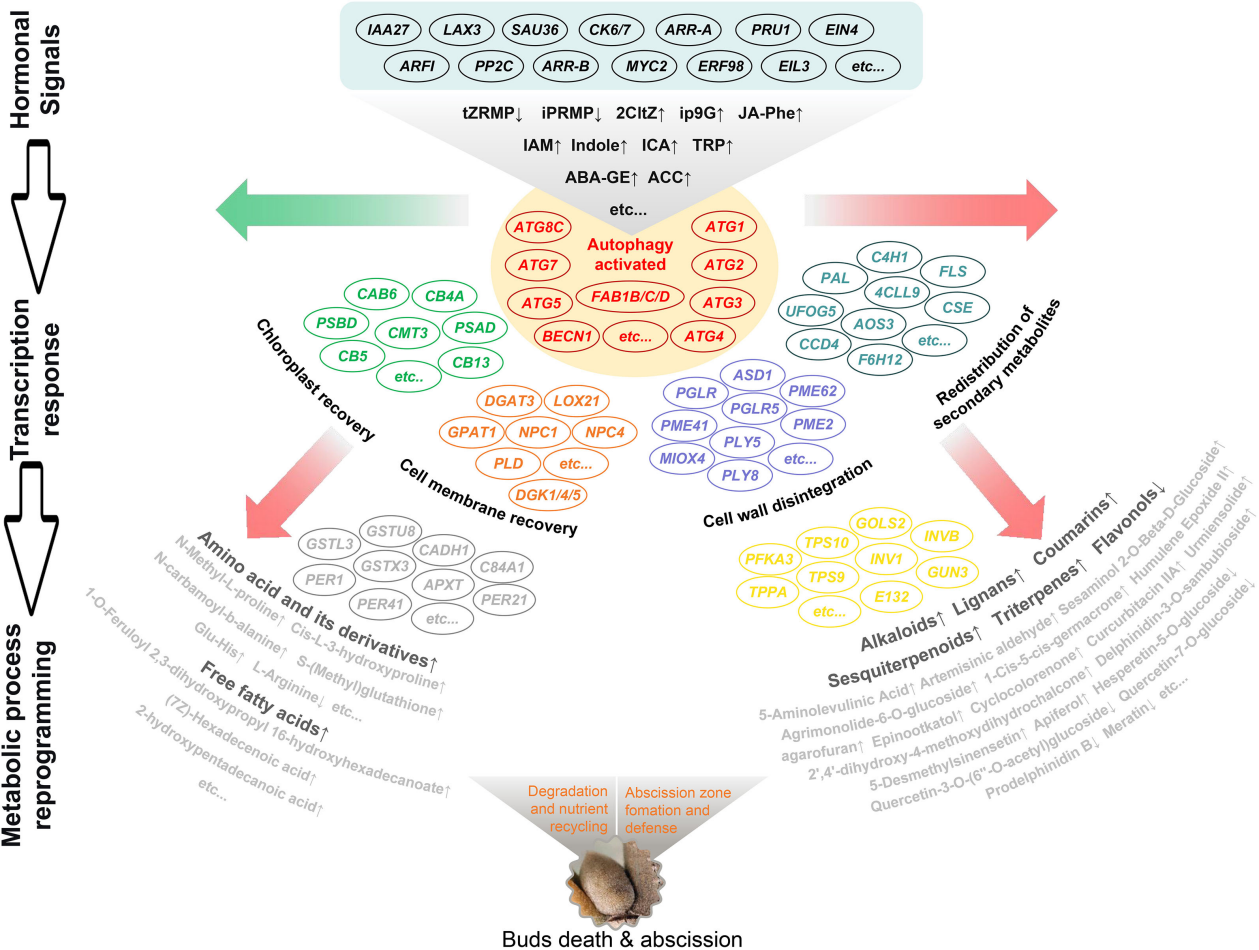
Coordinated regulation of hub genes and metabolites during chestnut bud PCD by an integrated multi-omics network (Representative connections are displayed.). Schematic diagram illustrating the hierarchical regulatory relationships during PCD. From top to bottom: phytohormone signaling, transcriptional responses associated with PCD, and metabolite reprogramming. Arrows indicate the direction of regulatory flow.

To systematically elucidate the molecular and metabolic basis of programmed cell death (PCD) in chestnut buds, we propose a three-layered regulatory model (**Figure 8**). At the hormone signaling level, enhanced ABA and ethylene signals (ABA-GE↑ and ACC↑), coupled with diminished cytokinin signaling (tZRMP↓), provide the upstream instruction that initiates the abscission program. At the transcriptional response level, these hormonal cues activate the expression of genes involved in phenylpropanoid metabolism (*PAL*, *4CL*, and *CAD*), cell wall modification (*PGLRs* and *PMEs*), and defense responses (*GSTs* and *PERs*). At the metabolic reprogramming level, changes in gene expression ultimately lead to the accumulation of defense-related metabolites, including phenylpropanoids, terpenoids, and alkaloids as well as remodeling of cell wall components. Together, these coordinated transcriptional and metabolic reprogramming events constitute a tightly regulated PCD process.

### 3.7 RNA-seq expression validation via qRT-PCR

**Figure 9:**
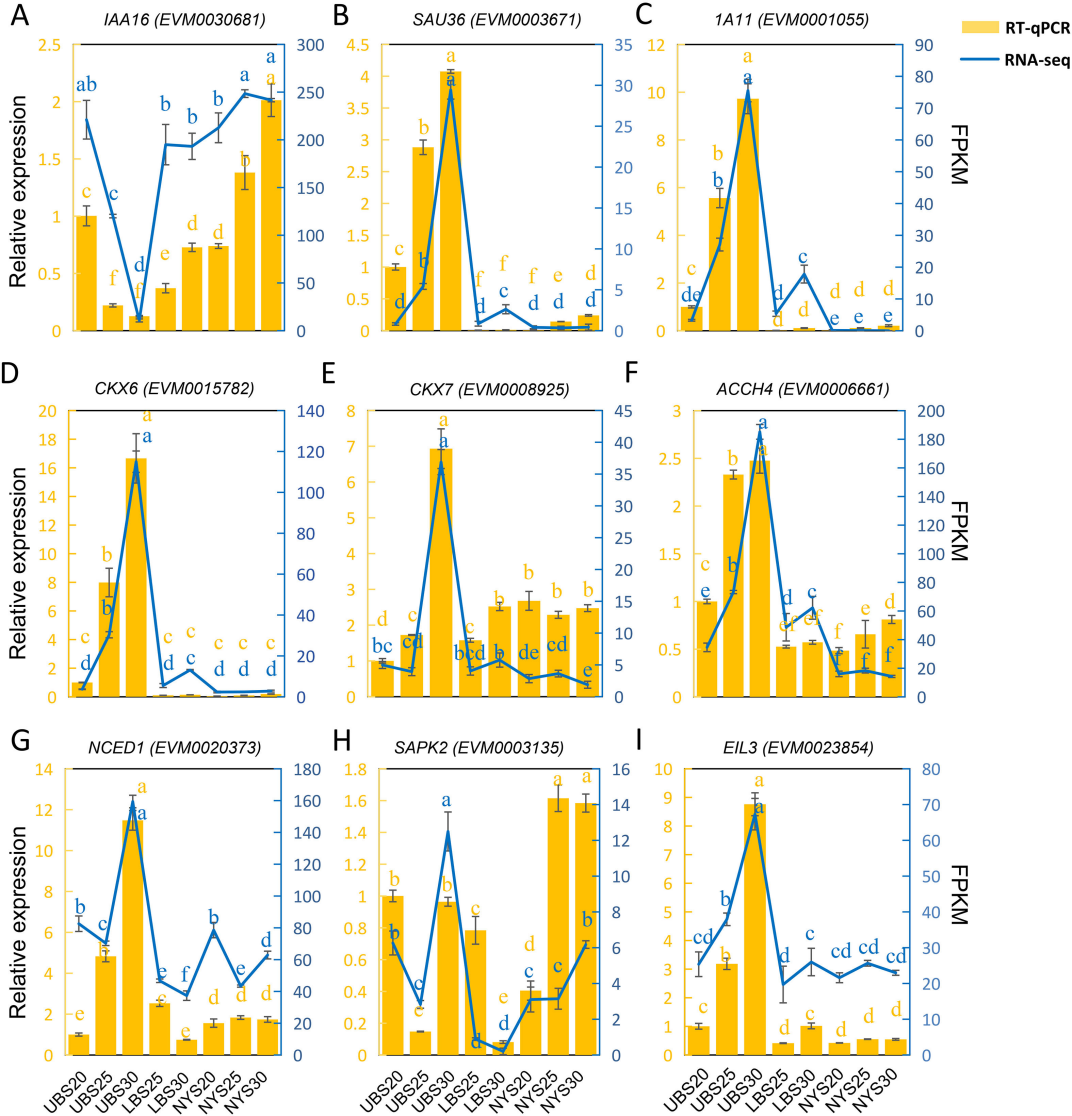
Validation of nine gene RNA-seq data by quantitative real-time PCR. **(A)** IAA16, **(B)** SAU36, **(C)** 1A11, **(D)** CKX6, **(E)** CKX7, **(F)** ACCH4, **(G)** NCED1, **(H)** SAPK2, and **(I)** EIL3. The relative expression levels determined by qRT-PCR (yellow bars, calculated using the 2^−ΔΔCT^ method) are shown alongside the normalized expression values (FPKM) from RNA-seq (blue line). Error bars represent the standard deviation of three biological replicates. Lowercase letters were determined based on the *P* < 0.05 calculation of the LSD method.

To validate the reliability of the RNA-seq data, we performed qRT-PCR on a set of nine hormone-related differentially expressed genes (DEGs). These included two auxin-related genes (*EVM0030681* and *EVM0003671*), two cytokinin-related genes (*EVM0015782* and *EVM0008925*), two ABA-related genes (*EVM0020373* and *EVM0003135*), and three ethylene-related genes (*EVM0001055*, *EVM0006661*, and *EVM0023854*). The expression patterns determined by qRT-PCR closely matched those from the RNA-seq analysis (**Figure 9**), confirming the reliability of the transcriptomic dataset.

## 4 Discussion

In this study, we employed an integrative multi-omics approach to elucidate the molecular regulatory network underlying programmed cell death (PCD) in chestnut buds, with a focus on the coordinated actions of phytohormone signaling, transcriptional regulation, and metabolic remodeling during bud abscission. Cytological observations revealed characteristic histological features of PCD, including tonoplast rupture, organelle degradation, nuclear condensation, and eventual cell-wall collapse (**Figure 2**), consistent with previous reports in chestnut (Wang et al., 2012). These cytological changes differ from the secondary cell-wall thickening and lignification typical of PCD in tracheary elements (Bollhoner et al., 2012), suggesting that PCD in different organs may employ distinct organelle degradation strategies tailored to their specific functions. The structural features observed here closely resemble those described during abscission of senescing organs such as petals and fruit stalks (Yamada et al., 2006; Van Doorn and Woltering, 2008; Ito and Nakano, 2015), providing a referenced context for constructing the transcriptional-metabolic regulatory network during PCD in chestnut buds.

In the present study, vacuole rupture identified in UBS25 was a critical event in the late stage of PCD, aligning with the “vacuole-mediated cell death” model in which the vacuole releases hydrolases that contribute to organelle degradation (Van Doorn and Woltering, 2008). Vacuolar rupture is often preceded by the dissipation of mitochondrial membrane potential and a burst of reactive oxygen species (ROS). In chestnut bud PCD, co-expression and protein-protein interaction analyses revealed the activation of an oxidative-redox homeostasis cluster comprising 67 genes, of which 45 were upregulated, and 22 were downregulated (**Supplementary Table 12**). In this study, we observed significant up-regulation of *PER1*, *PER4*, and *PER61*, whereas *PER9*, *PER17*, *PER19*, *PER49*, and *PER72* were markedly down-regulated. The peroxidase gene family has been reported to play a dual role during programmed cell death (PCD): on one hand, it scavenges excess H_2_O_2_ to mitigate oxidative damage; on the other, it participates in the utilization of H_2_O_2_ required for lignin polymerization (Zhang et al., 2022; Li et al., 2023, 2024). Accordingly, the up-regulation of *PER1/4/61* might be associated with lignification or the suppression of a hypersensitive like response, while the down-regulation of *PER9/17/19/49/72* could permit localized H_2_O_2_ accumulation to function as a signaling molecule activating downstream defense genes. This expression pattern reflected the fine-tuned regulation of redox homeostasis during plant PCD.

Phytohormones are among the most critical endogenous signals determining the initiation of senescence. In this study, hormone quantification showed that during bud PCD, the accumulation of active cytokinin forms (e.g., tZRMP, iPRMP) decreased, whereas JA-Phe, Phe, the storage form of abscisic acid (ABA-GE), and the ethylene precursor ACC accumulated significantly (**Figure 3F**). Marked accumulation of IAA-related and alkaloid biosynthesis precursors such as IAM, TRP, and Indole suggests a dual regulatory role for auxin in buds (Müller and Sheen, 2008; Sánchez-Parra et al., 2021). In the transcriptomic data, the lightcyan module from WGCNA contained 135 genes associated with hormone signal transduction, many of which were induced to varying degrees (**Supplementary Table 12**). This finding aligned with the conclusion of the previous study that phytohormone signaling was a central pathway in chestnut bud PCD (Guo et al., 2023). Extensive studies have shown that auxin and cytokinins could delay leaf senescence (Danilova et al., 2020; Song et al., 2025), whereas JA and ethylene promote leaf senescence (Huang et al., 2017; Nowshahri, 2024). This hormone dynamics pattern, i.e., attenuated growth-promoting signals coupled with enhanced senescence/stress signals, fits the typical profile of organ senescence and abscission (Sarwat et al., 2013; Guo et al., 2021; Lone et al., 2025). Thus, in the present study, the auxin pathway likely initially acted as a signal amplifier, with *SAUR* genes being rapidly induced. Subsequently, its core signaling was quickly subjected to negative feedback regulation (down-regulation of *TIR1*, *ARFs*, and *IAAs*) to prevent excessive growth promotion. In parallel, growth-promoting cytokinin signaling was suppressed, whereas JA/SA/ET pathways were activated and phenylalanine accumulated, leading to the initiation of a coordinated senescence program.

In this study, regulatory networks constructed from the lightcyan and saddlebrown co-expression modules enabled the identification of several PCD-associated functional clusters, particularly those involved in phenylpropanoid metabolism, glutathione metabolism, and cell wall modification. Within the phenylpropanoid biosynthesis cluster, phenylalanine ammonia-lyase (PAL) emerged as a hub gene with the highest connectivity. PAL catalyzes the first committed step in phenylpropanoid metabolism—the deamination of phenylalanine to cinnamic acid (Zhang and Liu, 2015). Up-regulation of *PALY* genes (*EVM0012757*, *EVM0026346*, and *EVM0031164*) aligned with the accumulation of downstream phenolic compounds in the metabolome, suggest that *PALYs* may act as a metabolic switch redirecting carbon flux toward defense-related secondary metabolites during bud PCD. GSTs were identified as core components of a detoxification and stress-response module. GSTs not only catalyze the conjugation of glutathione to electrophilic substrates but also participate in flavonoid vacuolar sequestration and stress signaling (Mueller et al., 2000; Wu et al., 2014; Ugalde et al., 2021). Up-regulation of *GSTU8* and its homologs (*GSTUA*, *GSTZ*, and *GSTL3*), together with accumulation of S-methylglutathione in the metabolome, pointed to a reprogramming of glutathione metabolism. This may function to protect cells from toxic byproducts generated during PCD and to modulate redox homeostasis. Polygalacturonases (PGs) are core hydrolases involved in pectin degradation, catalyzing the hydrolysis of α-1,4 glycosidic bonds in polygalacturonic acid (Anderson and Pelloux, 2025). Pectate lyases (PLs) act via *β*-elimination to cleave the same glycosidic bonds, functioning either synergistically or complementarily with PGs (Christiaens et al., 2016). Within the PPI network, up-regulation of genes encoding *PGLRs*, *PLY8*, and *PLY5* suggested the formation of a coordinated cell wall degradation module, providing the enzymatic basis for physical bud abscission.

In plants, the breakdown of the chloroplast is marked by thylakoid membrane disintegration and chlorophyll degradation—a process particularly evident in the senescent PCD of photosynthetic tissues such as leaves (Bosch and Franklin-Tong, 2024). Transcriptomic analysis revealed that genes related to photosynthesis and photosynthetic antenna proteins were significantly down-regulated in the late PCD stage (S30), indicating a complete shutdown of the photosynthetic system before bud abscission. This pattern aligns with observations in other plant senescence processes (Pružinská et al., 2005; Domínguez et al., 2021; Tanaka and Ito, 2025). Notably, within the network associated with chlorophyll degradation, the expression of a *DNA (cytosine-5)-methyltransferase CMT3* (*EVM0029690*) was significantly up-regulated during PCD in the present study (**Figure 7**). Some recent studies showed that the transcription of chlorophyll catabolic genes can be modulated epigenetically (He et al., 2018; Li et al., 2022), and the CHG methylation maintained by chromomethylase 3 (CMT3), that played a key role in regulating gene transcription, transposon silencing, and genome stability (Nozawa et al., 2021; Fang et al., 2022). Therefore, *CMT3* might play a critical role in actively down-regulating photosynthesis and promoting chlorophyll degradation during PCD in chestnut buds, which could be further tested by exogenous application of 5-Aza *in vivo*.

Transcriptomic and protein–protein interaction analyses revealed pronounced activation of the autophagy pathway (ko04136) during PCD in the upper buds of ‘Tima Zhenzhu’, providing molecular corroboration of the autophagic features. Key autophagy-related genes, including those encoding phosphatidylinositol 3-kinase (*PI3K*), target of rapamycin (*TOR1*), and multiple *ATG* family members, were significantly up-regulated (**Figure 7**). Studies indicated that *PI3Ks* participate not only indirectly in cellular signaling and vesicle trafficking, but also directly in autophagosome formation and autophagic flux (Li and Vierstra, 2012; Liu et al., 2018; Safaroghli-Azar et al., 2023). The TOR kinase senses nutrient status in plants and transmits signals to ATG13, a regulatory subunit of the upstream ATG1/ATG13 kinase complex, thereby modulating autophagy initiation (Feng et al., 2025). Autophagy receptors could directly recruit ATG proteins into receptor complexes, which in turn attract regulatory or core autophagy components to facilitate autophagosome assembly (Veljanovski and Batoko, 2014). Most *AtATG* knockout or knockdown mutants displayed phenotypes such as shortened life span, precocious senescence, altered stress tolerance, constitutive immune activation, and metabolic reprogramming even under standard growth conditions (Doelling et al., 2002; Chen et al., 2016). Thus, through the coordinated action of autophagy-related pathways, autophagosomes could deliver damaged organelles, proteins, and other cytoplasmic material to the vacuole for degradation, promoting resource recycling and contributing to cellular homeostasis during PCD of chestnut bud.

Although this study has revealed several insights, certain limitations should be acknowledged. First, the investigation was conducted using two specific cultivars (‘Tima Zhenzhu’ and ‘Dabanhong’); therefore, the conclusions should be extrapolated to other chestnut cultivars with caution. Second, while the multi-omics data uncovered a wealth of correlations, many of the functional inferences rely on predictions derived from studies of senescence in other plant organs. The causal relationships and functional roles of the key regulatory nodes identified here still require further validation through experimental approaches such as genetic transformation and gene editing.

## 5 Conclusion

In this study, a comprehensive investigation was performed on PCD buds of the Chinese chestnut through multi-omics integration analysis. Cytological observations revealed that the process was accompanied by hallmark autophagic features, including tonoplast rupture, organelle degradation, nuclear condensation, and cell-wall breakdown. Mult-omics profiling further indicated that these structural alterations were likely driven by hormonal reprogramming, characterized by increased levels of auxin precursors, decreased active cytokinin levels, and the accumulation of abscisic acid and ethylene. Subsequently, cells extensively up-regulated autophagy-related genes and activated redox systems to maintain homeostasis and support catabolic processes. In parallel, metabolic flux was redirected toward several secondary pathways, including amino acid and derivative metabolism, alkaloid biosynthesis, phenylpropanoid and lipid metabolism, as well as coumarin and terpenoid biosynthesis. This work provides the overall systematic link between the microscopic cytological signatures of PCD and the hormonal, transcriptional, and metabolic networks. The integrated model established here not only advances the understanding of organ abscission as a developmental process in plants but also offers a theoretical foundation and identifies a set of potential molecular targets for genetically shaping chestnut tree architecture.

## Data Availability

The datasets presented in this study can be found in online repositories. The names of the repository/repositories and accession number(s) can be found in the article/**Supplementary Material**. The raw sequencing data generated in this study can be found in the SRA (https://www.ncbi.nlm.nih.gov/sra/) of NCBI under accession number PRJNA1356349.
