# Supplementary material for: Combined hormonomic, transcriptomic, and metabolomic analyses reveal the role of programmed cell death in bud abscission of Chinese chestnut (Castanea mollissima BL.)
Source: Front Plant Sci. 2026 Apr 7;17:1781873. doi: 10.3389/fpls.2026.1781873 (PMC13097051; doi:10.3389/fpls.2026.1781873)
Supplement: Supplementary file 1 [file DataSheet1.docx]

Supplementary Material

# Supplementary Figures

**NYS20**

**NYS25**

**NYS30**


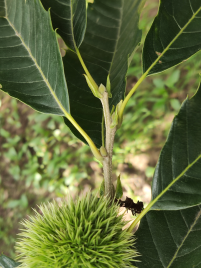

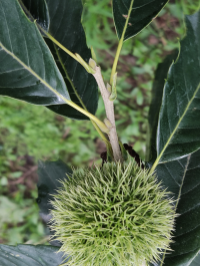

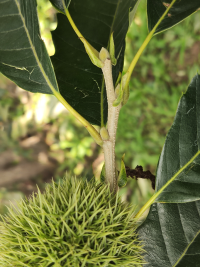

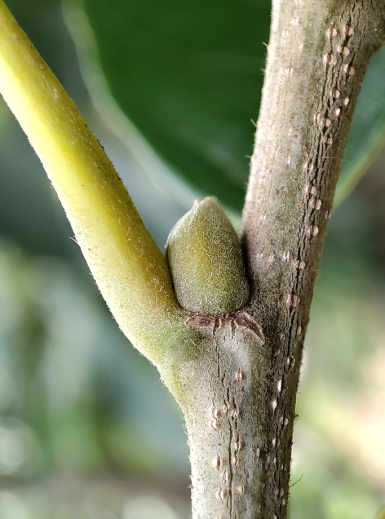

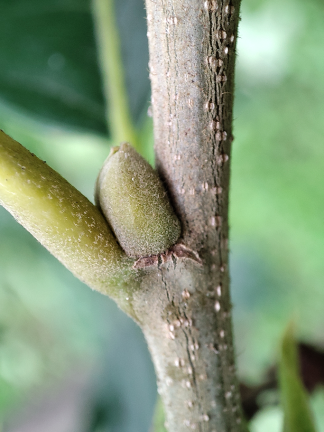

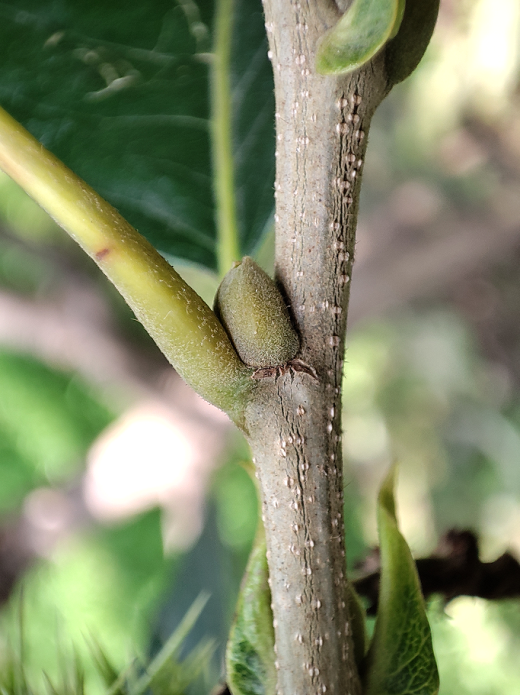


**B**

**A**


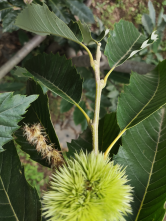


6 mm


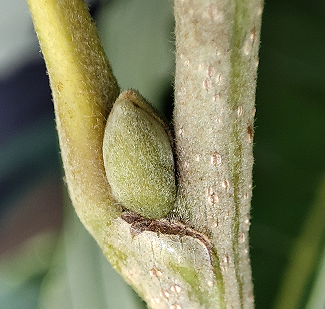


6 mm


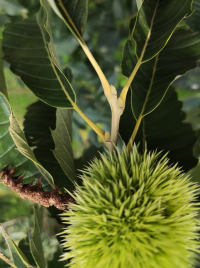


6 mm


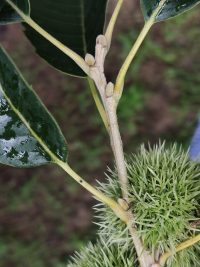


6 mm


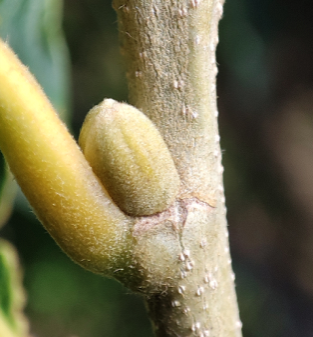


6 mm


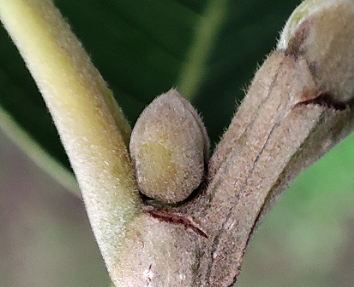


6 mm

**UBS20**

**UBS25**

**UBS30**

**C**


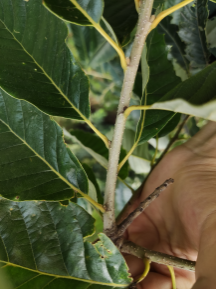


3.mm


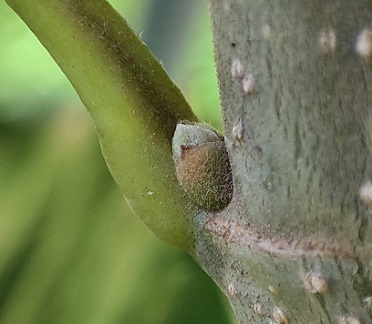


3 mm


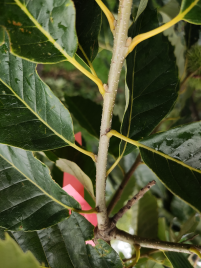


3.mm


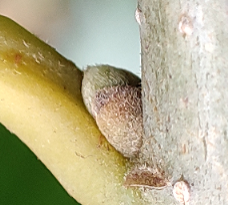


3mm

**LBS25**

**LBS30**

**Supplementary Figure 1.** Morphology of buds in chestnut. (A) The upper buds of 'Tima Zhenzhu'; (B) the lower buds of 'Tima Zhenzhu'; (C) the upper buds of 'Dabanhong'.


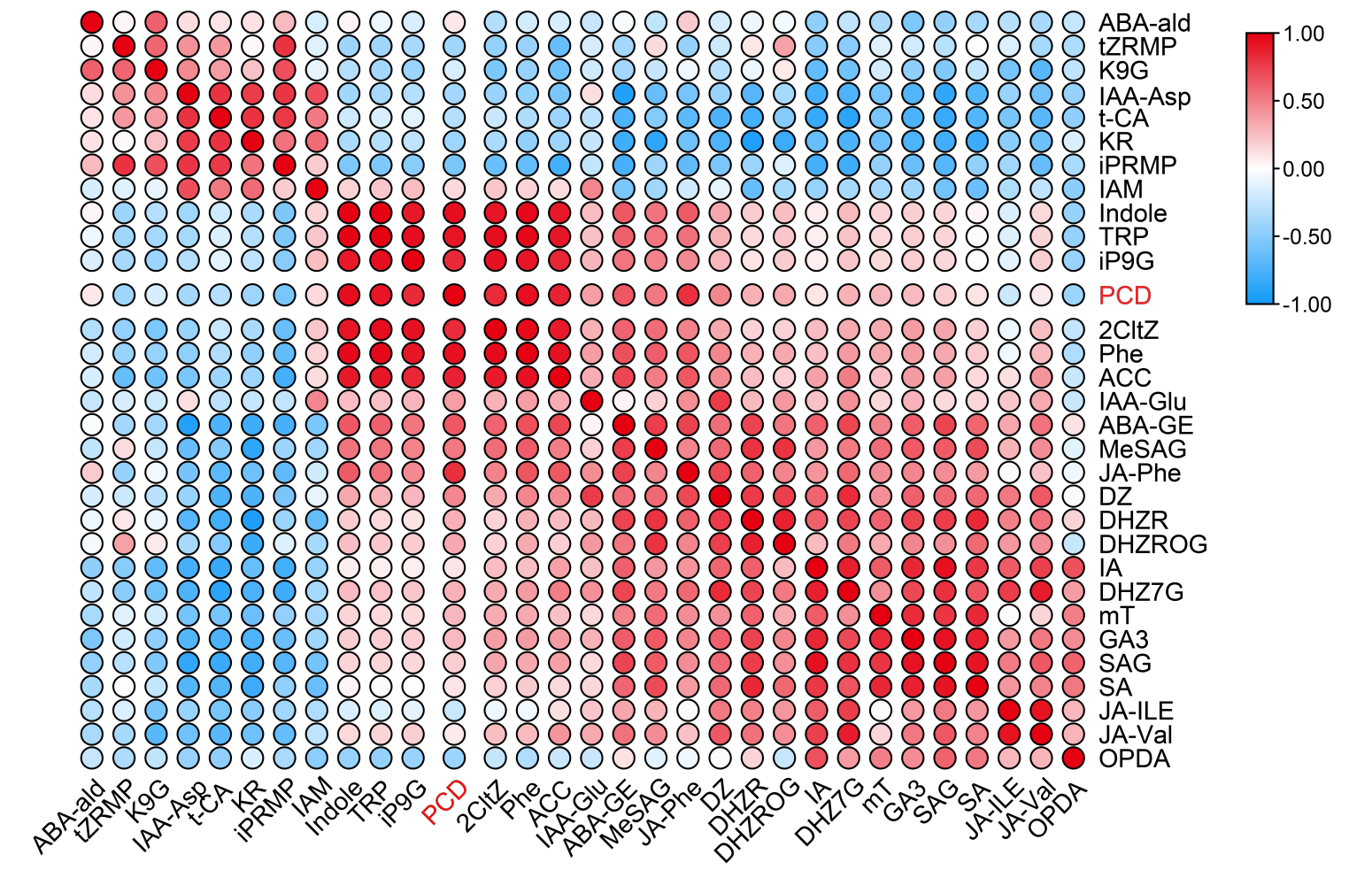


**Supplementary Figure 2.** Pearson correlation analysis between the PCD process and DAHs.


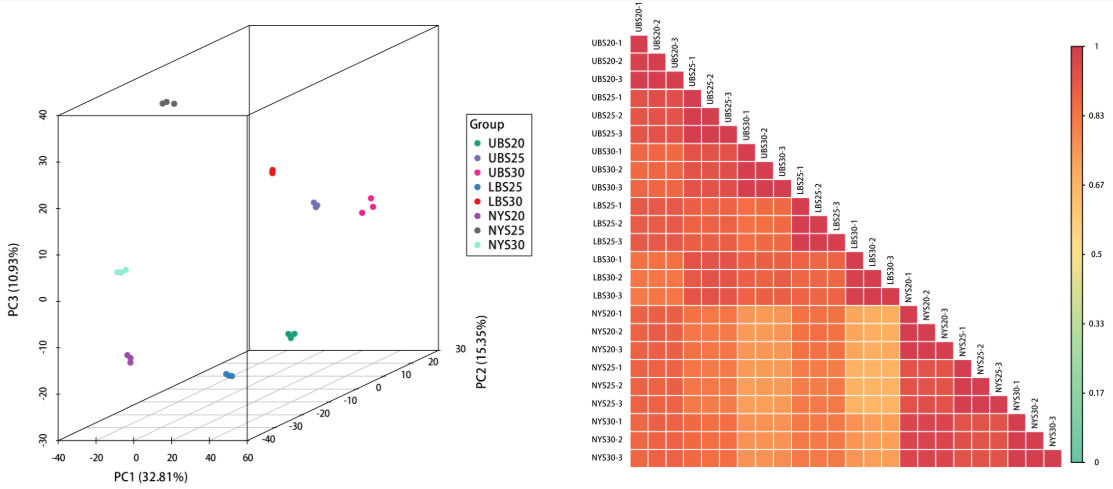


**Supplementary Figure 3.** Quality control analysis of 24 buds of metabolome. (A) PCA. (B) Pearson correlation.


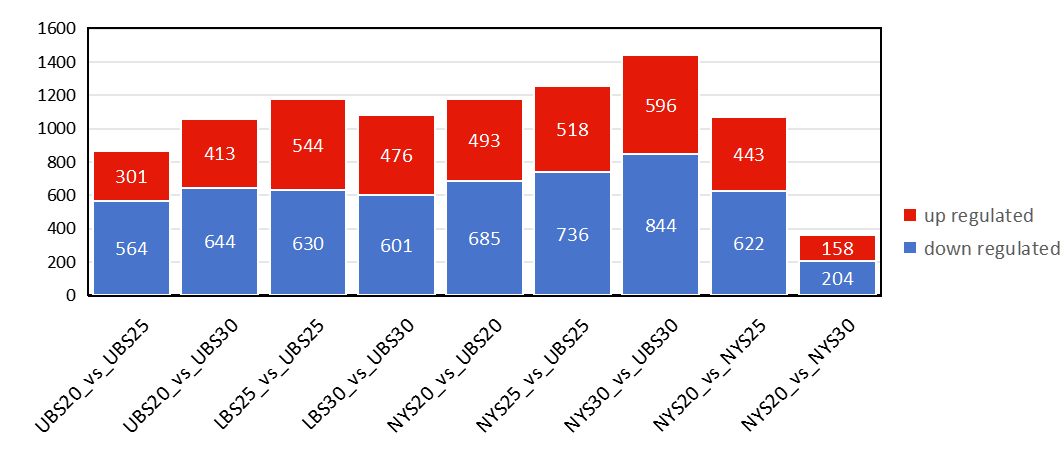


**Supplementary Figure 4** Differential metabolites identified in 24 samples. Red represents up-regulated, and blue represents down-regulated.


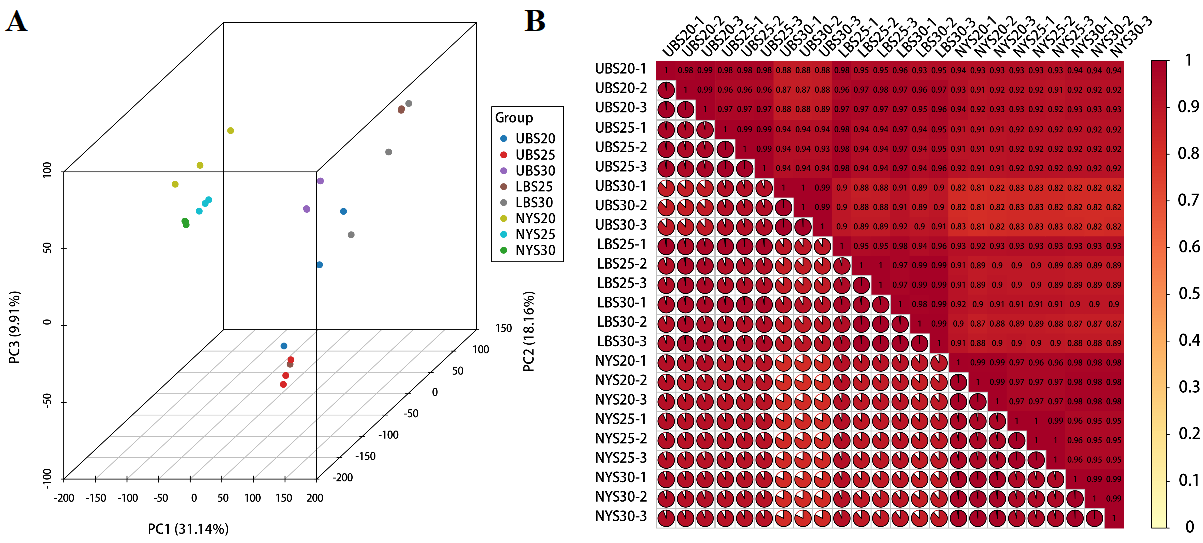


**Supplementary Figure 5** Quality control analysis of 24 buds of RNA-seq. (A) PCA. (B) Pearson correlation.


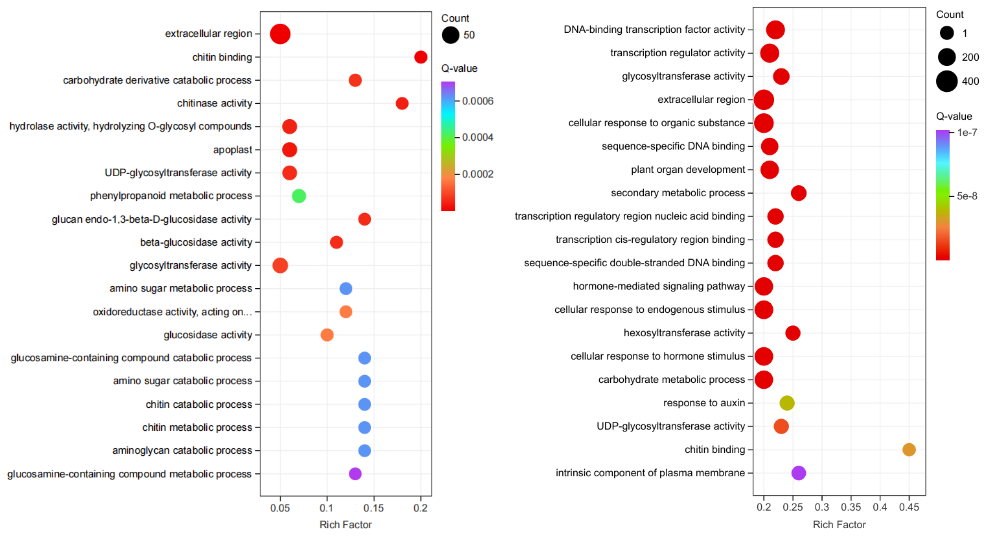


**Supplementary Figure 6** Enrichment analyses of the DEGs by GO term annotations. DEGs shared among (A) CK v.s. the S25 stages and (B) CK v.s. the S30 stages


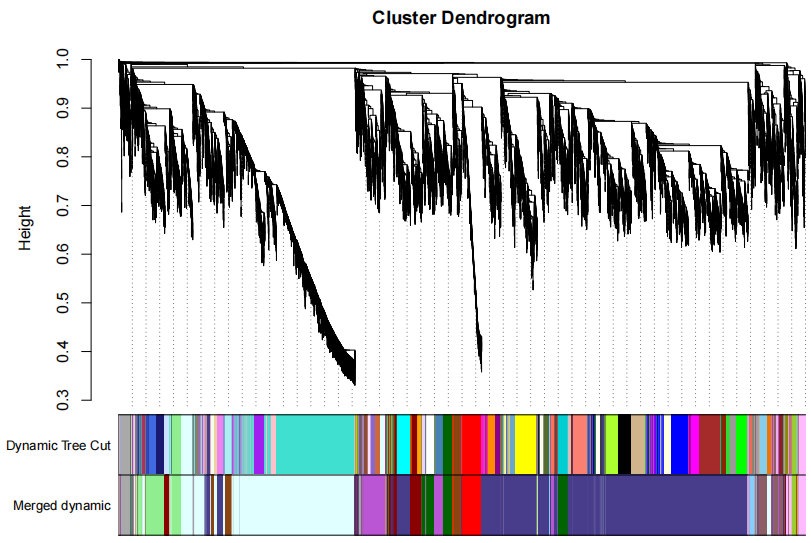


**Supplementary Figure 7** WGCNA gene clustering and module division.


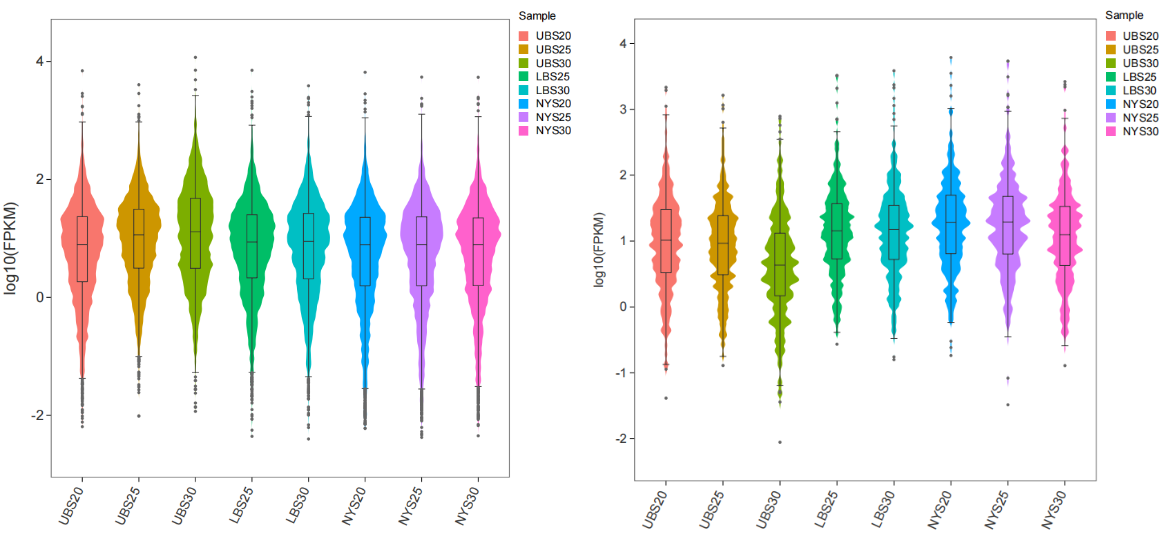


**Supplementary Figure 8** Expression patterns of genes in the lightcyan and saddlebrown modules. Gene expression profiles for the lightcyan (A) and saddlebrown (B) modules. The x-axis indicates sample names, and the y-axis represents log10(FPKM) values.
